# Battery-Free Tattooing Mechanism-Based Functional Active Capsule Endoscopy

**DOI:** 10.3390/mi13122111

**Published:** 2022-11-29

**Authors:** Manh-Cuong Hoang, Jong-Oh Park, Jayoung Kim

**Affiliations:** Korea Institute of Medical Microrobotics, Gwangju 61186, Republic of Korea

**Keywords:** active capsule endoscope, tattooing function, gastrointestinal tract diagnosis

## Abstract

This paper presents a novel tattooing capsule endoscope (TCE) for delivering a certain amount of ink to the submucosal layer of digestive tract organs. A dual-function permanent magnet is used for locomotion and injection activation. The developed capsule endoscope can move actively in 5 DOF due to the interaction between the permanent magnet and a controllable external magnetic field produced by an electromagnet actuation system. In addition, the permanent magnet is involved in a specially designed mechanism to activate a process that creates a squeezing motion to eject the liquid from the storage room to the target. The dimension of the prototype is 12.5 mm in diameter and 34.6 mm in length. The proposed TCE is tested ex vivo using a fresh porcine small-intestine segment. We were able to direct the TCE to the target and deliver the tattoo agent into the tissue. The proposed mechanism can be used for drug delivery or lesion tattooing, as well as to accelerate the realization of the functional capsule endoscope in practice.

## 1. Introduction

The gastrointestinal tract is home to many common and daunting diseases, especially colorectal cancer and stomach cancer, the third and fifth most common malignant tumors and the second and fourth leading causes of cancer-related deaths worldwide, respectively [1,2,3,4]. The incidence of patient admission related to the digestive tract is increasing due to the environment, food, and stress in life [1,5]. However, due to their lack of vision and physical touch, many disorders are difficult for clinicians to diagnose and treat. Early disease detection gives doctors better treatment options, increasing their ability to treat patients and extend their lives.

Laparoscopic surgery is a minimally invasive surgery technology for the treatment of digestive diseases, in which a segment of infected organs with ulcers is removed [6,7]. This technique is in demand due to its promise of less pain, a shorter hospitalization time, and advanced long-term cosmetics [8]. In order to identify a target area for surgical treatment during laparoscopic surgery, the localization of lesions during preoperation is an essential step [9]. Poor identification could result in a longer procedure and even the removal of the wrong portion of the bowel [6,10,11]. Accurately marking the biopsied site is also crucial to monitor chronic atrophic gastritis. A safe and efficient approach to aid in surgical localization is the endoscopic tattooing of colonic lesions, where the tattooing agent is used to dye the tissue for visual recognition using an endoscopic instrument [9,12]. However, using an endoscopic device could cause the patient to feel uneasy; even worse, it may induce sedation-related side effects or fear in the patient. Moreover, this tool may cause pain due to stiffness and has limitations in high-curvature organs or deep positions. Needless to say, the reachable range of this tool is limited to the colon, and it cannot perform tattooing in the small bowel. The demand for noninvasive surgery is growing along with technological improvements, particularly in microelectromechanical systems.

In a noninvasive procedure known as wireless capsule endoscopy, a pill-sized robot called a wireless capsule endoscope (WCE) can passively move through the gastrointestinal tract to inspect the organ’s inner surface [13]. Due to its small size, this device can be ingested orally without causing any discomfort, fear, or side effects due to sedation. The data are obtained wirelessly by a wearable device, allowing for patients to go about their regular lives and jobs. Since being introduced in 2000, WCEs with remarkable advantages have been commercialized by several companies, such as Given Imaging (Israel) [14], Jinshan (China) [15], and Intromedic (South Korea) [16]. Passive motion, which causes blind zones that cannot be clearly monitored, greatly restricts this strategy. To overcome this difficulty, active WCEs with actuators that allow for capsule robots to move under control were developed [17,18]. After that, active WCE-integrated functional modules were widely researched to expand this technology’s capabilities, including biopsy, drug delivery, and tattooing. Several research groups presented biopsy modules for capsular endoscopes that could wirelessly extract sample tissue [19,20,21]. In a different application, a WCE can be used in conjunction with a drug-delivery module to treat gastrointestinal disorders [22,23,24,25]. There are many studies on these two functions in the literature, but very few on tattooing. Preoperative tattooing is crucial for laparoscopic surgery; the integration of such a function for WCEs has not been thoroughly researched due to substantial obstacles such the small size of the capsule body, the force/pressure for injection ink, and needle control. With the goal of addressing the limitations of conventional endoscopic tattoos and expanding the application for capsule endoscopes, capsule endoscope-integrated tattooing functions were proposed for dyeing tissue in digestive organs [26,27,28,29]. The developed functioning capsule robot can move actively and release the tattooing agent at the target. However, these designs are limited by the activation mechanism. It must be powered by electricity, which is not desirable due to the small capacity of onboard cell batteries. Several drug-delivery capsule endoscopes for which injection mechanisms were proposed are relevant to this study [30], such as the gas-based injection mechanisms controlled by electronic circuits [31,32], microthruster [33], gas-generated chemical reaction [34,35], and motorized drug delivery [36,37]. In this paper, we propose a complete solution for a tattooing capsule endoscope (TCE) that can actively move in the digestive tract and perform tattooing with a battery-free mechanism. The TCE design began with a few specifications to solve these difficulties, as follows:

- First, the robot utilizes the advantages of a WCE that can move actively in the GI tract. The capsule should be able to direct the injection needle toward the target, which is the area surrounding the organs. The magnetic force from the controlled system should be enough for the needle to penetrate the tissue. During locomotion, the injection mechanism should be disabled. Upon reaching the target, it will be triggered to eject ink beneath the mucosa layer.
- Second, the capsule can regulate the needle’s condition to address safety concerns. To prevent damage, and even perforation, during movement, the tattooing needle should not be exposed to the exterior of the capsule body. The capsule can protrude the needle at the target to pierce the mucosa layer of the organ and withdraw once the tattooing is complete.
- Third, the ejection mechanism should generate an adequate force to expel the ink from the container into the tissue. In this study, we focus on using chemical reactions that can create pressure to push a piston. The resultant force is controllable due to the reactant content. Gas-generation chemical reactions can lead to high pressure with a low volume of reacting substances. Therefore, we can minimize the size of the tattooing module and the capsule robot.
- Fourth, a battery-free triggering mechanism is desired. Since the capacity of a battery embedded inside a capsule body is limited to the energy for the camera and wireless communication, the battery-free design is a perfect solution to the long-term operation of the capsule robot.

The result of this design effort is a tattooing capsule endoscope with a chemical reaction-based injection force. This work proposes a battery-free tattooing robotic capsule endoscope to mark target lesions in the small bowel and colon. The capsule is actively manipulated by an electromagnetic actuation (EMA) system for visual investigation and tissue tattooing. The proposed robot performs four main tasks in sequence for tattooing: needle extrusion, needle penetration, ink injection, and needle retraction. A cubic permanent magnet is used for both locomotion and tattooing activation. A mixture of two dry chemical powders is used to create a gas that pressurizes the propellant room to push the piston, injecting ink into the submucosa layer. The activation of the chemical reaction is controlled by the triggering mechanism using a magnetic force from the permanent magnet. A prototype with a pill-shaped capsule was fabricated and tested. Through basic tests and ex vivo experiments, positive results prove the feasibility and potential of the proposed robot. This paper is an extension of the previous research [38], with the following contributions: (1) A new design and mechanism for injection needle management, as the previous version showed limitations. Previously, the extrusion mechanism was performed by rotating the tattooing module and could be executed while moving or scanning. In this newly developed mechanism, we added a key that only allows for needle extrusion when its axis is collinear to the hole. This prevents unexpected extrusion of the injection needle during locomotion. (2) A redesigned triggering mechanism to break the water container. Since the size of the microneedle is small, at 0.1 mm, the water cannot passively exit the container. By adding a four-rod patch, we can actively squeeze water out, and thus increase the likelihood of triggering. (3) The magnetic actuation method with a newly developed control algorithm was designed using the optimization programming to increase the magnetic field and force and more powerfully control the robot. Recently, we proposed this control algorithm and demonstrated its controllability through closed-loop control of a magnet-based cube; this is the first application of this control strategy to a functional capsule endoscope.

## 2. Materials and Methods

### 2.1. Overall System and Design of TCE

**Figure 1:**
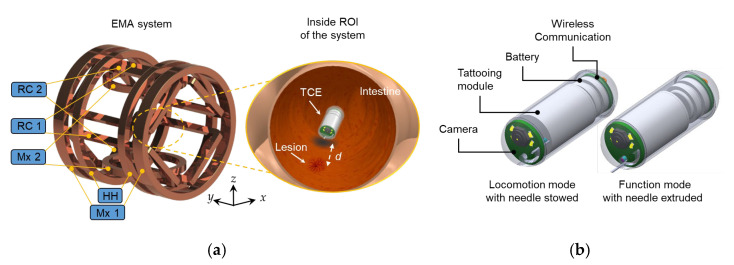
(**a**) Overall description of the TCE system and (**b**) conceptual design of the TCE in isometric view. *D* (=2 to 5 cm) is the recommended tattoo distance. Mx 1 is Maxwell coil aligned along the *y*-axis, Mx 2 is Maxwell coil aligned along the *z*-axis, HH is the Helmholtz coil aligned along the *y*-axis, RC 1 and RC 2 are customized rectangular coils arranged in four quarters of the *xz* plane.

Figure 1a provides an overview of the TCE system, consisting of the EMA system and a TCE. The EMA system is composed of five pairs of coils, including Maxwell coil 1, Maxwell coil 2, a Helmholtz coil, and two pairs of rectangular coils. The specifications of the EMA system are depicted in Table 1. The EMA system generates a magnetic field to control the TCE inside the region of interest (ROI) of the system. During interaction between the permanent magnet and the external magnetic field, the TCE can actively travel in the digestive tract and mark the targeted lesion by injecting a long-lasting agent. For the locomotion function, the TCE is designed to be controlled in five degrees of freedom, including moving in the *x*, *y*, and *z* axes and rotating pitch and yaw motions. With the 5 DOF motion, the TCE can guarantee a thorough scanning without a blind area. The TCE is switched to function mode when a lesion or abnormality is detected. Here, the TCE uses a needle to penetrate the submucosal layer and accurately deliver the tattooing material. It is recommended that the tattooing spot ranges from around 2 to 5 cm from the targeted object.

Figure 1b shows a conceptual design of the TCE consisting of four main parts: a miniature camera module, a tattooing module, a battery pack, and a wireless communication module. The battery powers the camera, and the image frames are transmitted through the wireless communication module to an external acquisition device. The tattooing module is devised as a battery-free mechanism with a medical function to deliver a tattooing agent at the desired location. The TCE works in two modes, locomotion and function mode. In locomotion mode, the needle is stowed inside the capsule body to prevent tissue damage. In contrast, in function mode, the tattooing needle is forced out of the capsule body to enable the tattooing process. In this study, the tattooing agent is India ink diluted with saline water at a ratio of 1:100. The needle penetration depth to the tissue is 1 mm beneath the mucosa layer, which guarantees long-lasting tattooing.

### 2.2. Design of the Tattooing Module

**Figure 2:**
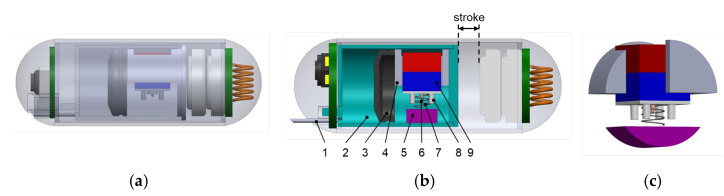
(**a**) Transparent view of the tattooing module inside capsule shell. (**b**) Detailed design of the tattooing module with components: 1, injection needle; 2, module body; 3, rubber piston; 4, permanent magnet guidance; 5, water reservoir; 6, microneedle; 7, helical spring; 8, four-rod pad to squeeze water; 9, cubic permanent magnet. (**c**) Isometric view of the water squeezing module.

As mentioned earlier, the injection force used to deliver the ink into tissue derives from a chemical reaction between two powders: citric acid and sodium bicarbonate. The reaction between these substances generates carbon dioxide gas, whose concentration increases over time. The increase in gas volume results in a pushing force on the piston. Once this force exceeds the static friction force between the piston and the surface, the piston will be pushed forward, ejecting ink through the injection needle. Interestingly, the reaction between these two agents occurs when they come into contact with water, which allows for us to actively trigger the phenomenon by designing a mechanism that can splash water into the chemical mixture. Figure 2 shows the design of the tattooing module. The permanent magnet can move up/down, while the other motions are locked by the magnet guidance part. The chemical powders are mixed and stored inside the cavity of the piston. A total of 0.02 mL water is stored inside the water reservoir made of nylon. To trigger the chemical reaction, a magnetic force controlled by the EMA is generated to push the permanent magnet downward. When the magnetic force is greater than the reaction force of the spring, the microneedle will puncture the water reservoir. Since the microneedle’s size is tiny, with a size of 0.5 mm, water can barely be forced out. Therefore, we used a four-rod pad to create a squeezing force on the water tank. This facilitates a high likelihood of contact between reagents and water. In this design, a helical spring works as a safety key to prevent unintended activation. Based on the stiffness of the spring, we can estimate the required force for triggering, and thus can set a magnetic force threshold for safe movement.

### 2.3. Design of the Needle Management Module

**Figure 3:**
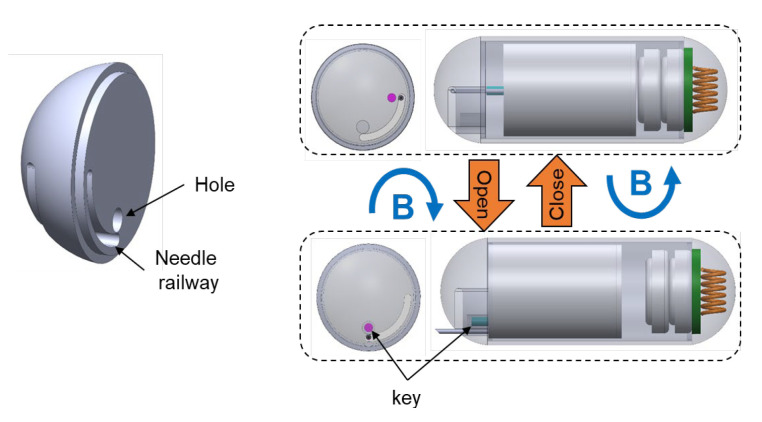
Design of the needle management module with the head dome (**left**) and control mechanism (**right**). The blue arrow indicates the rotation magnetic field direction B. A drawing of the head dome is shown in Appendix A.

The needle management mechanism is of great importance to avoid damage to the tissue, or even perforation in the worst case. Figure 3 shows the design and working mechanism of the needle management module. The tattooing module can slide forward and backward inside the capsule shell for needle extrusion and retraction, respectively. Since the head dome is in the way and prevents forward mobility, the key locks its linear motion. The key must be rotated 90 degrees clockwise to unlock both the injection needle’s linear motion and the key’s linear motion. The tattooing module can roll along with the permanent magnet’s rotation because it is not connected to the capsule’s outer shell. This rolling motion around the axial axis of the capsule body can be realized by the magnetic field produced by the EMA system. To allow for this rotation motion, the head dome is modified with a needle railway that facilitates the needle rotation within 90°. After rotating clockwise, the key and hole become concentric and enable the linear motion of the key and the injection needle. In this case, a magnetic force can be employed to extrude and retract the injection needle. In the extrusion status, the key is placed inside the hole. In contrast, a counterclockwise magnetic field is needed to rotate the key to the initial position, which is 90° away from the hole, following the withdrawing motion of the injection needle to lock its linear motion.

In this section, we present the overall system, design, and working principle of the tattooing module and the needle management module. In summary, the tattooing process consists of the following steps:

1. Travel to the destination;
2. Rotate clockwise two loops to enable needle extrusion;
3. Command a forward movement to expose the needle;
4. Insert the needle into the tissue;
5. Trigger the chemical reaction using magnetic force;
6. Wait for ink delivery;
7. Withdraw the needle and roll the TCE counterclockwise to lock the needle’s linear motion;
8. Travel to visual of the other area.

### 2.4. External Magnetic Field Control with 5 DOF Locomotion Motion

The functional capsule robot in this study is controlled by an external magnetic field generated by the EMA system, as depicted in Figure 1. The EMA system consists of ten stationary electromagnets, and thus, the magnetic torque and force can be derived by interaction between a magnet inside the CE and an external magnetic field as follows:

(1) $$\tau =\mu_{0}M\times H$$

(2) $$F=\mu_{0}\left(M\cdot \nabla \right)H=\mu_{0}{\left[\begin{array}{ccc} \frac{\partial H}{\partial x} & \frac{\partial H}{\partial y} & \frac{\partial H}{\partial z} \end{array}\right]}^{T}M\;$$

where $\tau \in {\mathbb{R}}^{3\times 1}$ (Nm) is the magnetic torque, $F\in {\mathbb{R}}^{3\times 1}$ (N) is the magnetic force, $M\in {\mathbb{R}}^{3\times 1}$ is the magnetic moment of the capsule robot, $H\in {\mathbb{R}}^{3\times 1}$ (A/m) is the magnetic field intensity, and $\mu_{0}=4\pi 10^{-7}$ (N/A^2^) is the vacuum permeability. Since the magnetic moment of the capsule robot is fixed, the way to control the magnetic torque and force is to employ the external magnetic field. The magnetic field in the ROI is uniform, and it can be expressed as the summation of the individual sources, as follows:

(3) $$H=\overset{m}{\underset{k=1}{\sum }}H_{k}=\overset{m}{\underset{k=1}{\sum }}{\bar{H}}_{k}i_{k}$$

where m is the number of electromagnets, and $H_{k}$ is the magnetic field of the *k*-th electromagnet, which is linearly proportional to the exciting current $i_{k}$ with the factor ${\bar{H}}_{k}\in {\mathbb{R}}^{3\times 1}$ (the magnetic field intensity at 1 A). The summation of the magnetic field can be expressed in algebraic form:

(4) $$H=\left[\begin{array}{ccc} {\bar{H}}_{1} & {\bar{H}}_{2} & \begin{array}{cc} \cdots & {\bar{H}}_{m} \end{array} \end{array}\right]\left[\begin{array}{c} i_{1}\\ \vdots \\ i_{m} \end{array}\right]=\bar{H}I$$

where $\bar{H}\in {\mathbb{R}}^{3\times m}$ is the unit–current magnetic field matrix, and $I\in {\mathbb{R}}^{m\times 1}$ is the current vector at electromagnetic coils. From Equation (4), the magnetic field gradient term in Equation (2) can be rewritten as follows:

(5) $$\frac{\partial H}{\partial x}=\left[\begin{array}{cc} \frac{\partial {\bar{H}}_{1}}{\partial x} & \frac{\partial {\bar{H}}_{2}}{\partial x} \end{array}\begin{array}{cc} \cdots & \frac{\partial {\bar{H}}_{m}}{\partial x} \end{array}\right]\left[\begin{array}{c} i_{1}\\ \vdots \\ i_{m} \end{array}\right]=\frac{\partial \bar{H}}{\partial x}I$$

(6) $$\frac{\partial H}{\partial y}=\left[\begin{array}{cc} \frac{\partial {\bar{H}}_{1}}{\partial y} & \frac{\partial {\bar{H}}_{2}}{\partial y} \end{array}\begin{array}{cc} \cdots & \frac{\partial {\bar{H}}_{m}}{\partial y} \end{array}\right]\left[\begin{array}{c} i_{1}\\ \vdots \\ i_{m} \end{array}\right]=\frac{\partial \bar{H}}{\partial y}I$$

(7) $$\frac{\partial H}{\partial z}=\left[\begin{array}{cc} \frac{\partial {\bar{H}}_{1}}{\partial z} & \frac{\partial {\bar{H}}_{2}}{\partial z} \end{array}\begin{array}{cc} \cdots & \frac{\partial {\bar{H}}_{m}}{\partial z} \end{array}\right]\left[\begin{array}{c} i_{1}\\ \vdots \\ i_{m} \end{array}\right]=\frac{\partial \bar{H}}{\partial z}I$$

Hence, the magnetic force in Equation (2) also can be expressed in algebraic form with respect to the input current, as follows:

(8) $$F=\mu_{0}{\left[M\begin{array}{ccc} \frac{\partial \bar{H}}{\partial x} & M\frac{\partial \bar{H}}{\partial y} & M\frac{\partial \bar{H}}{\partial z} \end{array}\right]}^{T}I$$

Assuming that the CE follows the magnetic field direction under a sufficient magnetic torque, the direction and position of the capsule can be controlled through the following governing equation:

(9) $$\left[\begin{array}{c} B\\ F \end{array}\right]=\mu_{0}{\left[\begin{array}{cc} H & M\frac{\partial \bar{H}}{\partial x} \end{array}\begin{array}{cc} M\frac{\partial \bar{H}}{\partial y} & M\frac{\partial \bar{H}}{\partial z} \end{array}\right]}^{T}I=AI$$

To utilize the redundancy of the EMA system, we applied the optimization-based control to obtain a high magnetic field and field gradient. Thus, the currents at the electromagnets can be solved by an optimization routine as follows:

Cost function:

(10) $$\min\;f=w\sqrt{I^{T}I}+\left(1-w\right)\sqrt{\frac{\sum {\left(i_{k}-mean\left(I\right)\right)}^{2}}{\left(m-1\right)}}$$

Subject to

(11) $$\left[\begin{array}{c} B\\ F \end{array}\right]=AI$$

(12) $$I_{min}\ll I\ll I_{max}$$

This cost function aims to find a solution that has a minimized mean and standard deviation to satisfy constraints Equations (11) and (12). As proven in [39], this optimization-based control strategy can extend the control range of the magnetic field and field gradient strengths, improve the control accuracy, and prevent the bias current. The needle control mechanism can be realized by continuously changing the magnetic field’s direction while maintaining its amplitude.

## 3. Results

### 3.1. Prototype Fabrication and Experiment Setup

**Figure 4:**
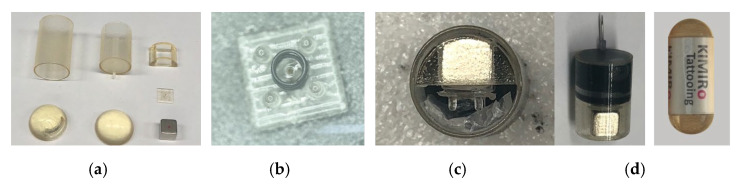
Fabrication of a TCE prototype: (**a**) 3D printing components; (**b**) assembled 4-rod pad with microneedle and helical spring; (**c**) assembled tattooing module with back view (**left**) and top view (**right**); (**d**) assembled TCE.

A prototype was fabricated to validate the feasibility of the proposed mechanism. Figure 4a shows the individual components of the prototype. The body cover, tattooing module, and magnet guide were printed using a 3D printer (Eden, Stratasys Direct Manufacturing Ltd., Valencia, CA, USA) using Veroclear material. The cubic permanent magnet was 5 mm × 5 mm × 5 mm N42 Neodynium, and the helical spring was 2 mm in length and 2 mm in diameter with 0.05 N/mm stiffness (purchased from Misumi, Seoul, South Korea). We used a rubber piston with dimensions of 9.7 mm diameter and 6 mm height from a commercial 3 mL syringe. A 26-gauge medical needle was used as the injection needle. Figure 4b,c show the assembled rod pad and tattooing module, respectively. The completed prototype, with a 12.5 mm diameter and 34.6 mm length, is illustrated in Figure 4d.

**Figure 5:**
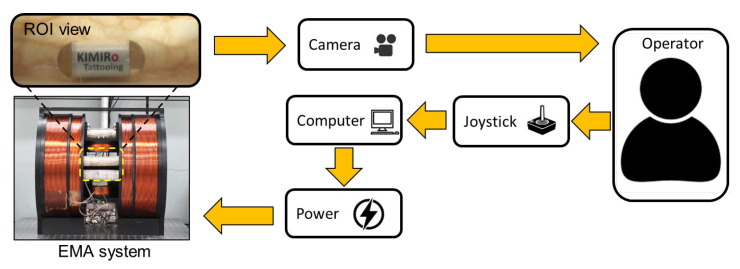
Scheme of experiment setup. The tattooing capsule was controlled by the magnetic field using a keyboard and a joystick. The controlled parameters were computed by the control software in the computer. The power supplies were used to provide a current to each coil using the control command via the software.

To evaluate the prototype’s performance, we set up the experiment as shown in Figure 5. The system was controlled by the human-in-loop control strategy. We used a camera (Canon, 600D, Tokyo, Japan), fixed outside the system, to record the movements of the robot in the *xz* plane. The operator controlled the robot’s motions using a joystick (Extreme 3D Pro Joystick, Logitech, Lausanne, Switzerland) with the feedback signal from the camera. The direction angle was controlled at an increment of 1°, while the magnetic field and gradient field were adjusted at resolutions of 1 mT and 0.05 T/m, respectively. The capsule was first aligned by the magnetic field and then pushed by the magnetic force. The rotation motion to switch modes was automatically performed by pressing a button on the joystick. The controlled parameters of the joystick were inputs of the control software, built in LabVIEW 2017 (National Instruments, Austin, TX, USA). The control algorithm was run in the central computer to compute the electrical currents of each electromagnet. The power supplies (MX15 five units and 3001LX five units from California Instrument, San Diego, CA, USA) were connected to a computer and controlled by a PCI-based GPIB controller to provide a current to each coil using the control command via the LabVIEW software.

### 3.2. Injection Force

**Figure 6:**
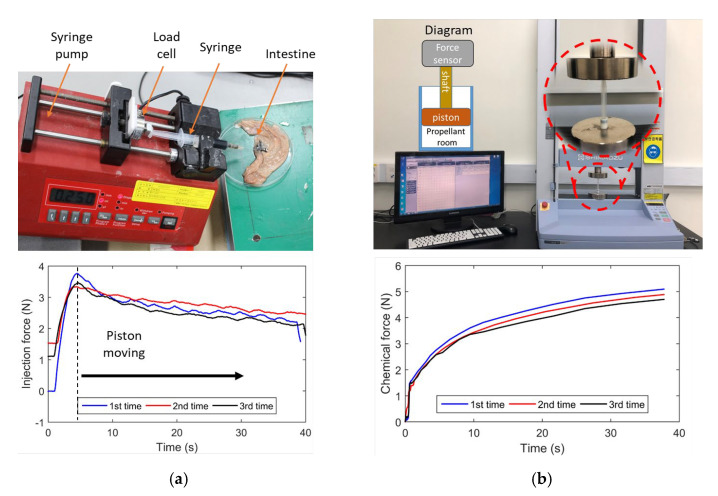
(**a**) Experimental setup to investigate the required force to deliver ink into the tissue of the pig’s colon (**top**) and results (**bottom**). (**b**) Experiment setup with a diagram to estimate the force on a piston generated by the chemical reaction (**top**) and results (**bottom**).

The production of carbon dioxide gas provides the force needed to move the piston. The force needed to inject ink into the submucosal layer must, therefore, be investigated in order to determine the quantity of chemicals that is required. The experimental setup needed to examine the injection force into the pig colon’s submucosal layer is shown in Figure 6a. The experiment used needles of 24, 25, 26, and 27 gauge at different sizes. Since the inner diameter of the 3 mL syringe matches that of the tattoo module, it was chosen for the tests. Ink was submucosally injected using a New Era Pump Systems NE-1000 Programmable Single Syringe Pump (Syringe Pump, Farmingdale, NY, USA). The syringe pump was mounted at a 30° angle to the horizontal. A pusher component of the syringe pump was attached to a Transducer Techniques GSO series load cell (Transducer Techniques, Temecula, CA, USA) with a 1000 g capacity. The load cell was linked to the TMO-2 load cell amplifier, which was then calibrated to have linear characteristics. A total of 0.2 mL of diluted India ink was pumped at a rate of 0.6 mL/min. Figure 6a shows the acquisition result with a sampling rate of 1 kHz. We can see that approximately 4 N was needed to overcome the static friction force. Once the piston moved, the injection force reduced due to the reductions in kinetic friction force and ink volume. Therefore, it was concluded that 4 N is the necessary pushing force to inject ink into the submucosal layer. The experiment was carried out in a temperature-neutral environment.

After estimating the necessary ink injection force, we determined the required reagent quantity using the ideal gas law. It is vital to verify the force that results from an increase in the volume of gas that is created because several elements, including temperature, solubility, and even the heat of formulation, affect the solution. We used a testing machine (Shimadzu AGS-x Series, Columbia, MD, USA) to monitor the real-time force from the chemical reaction acting on the piston. The piston was connected to the sensor of the machine, as shown in Figure 6b. A closed-capsule body, without the ink chamber but containing a propellant room, was used (see the diagram in Figure 6b). In the propellant chamber, 0.08 g of acid citric and 0.035 g of sodium bicarbonate were mixed and loaded. The chemical reaction was started by injecting 0.02 mL of water with a 30G-needle syringe via the sealing piston. As illustrated in Figure 6b, the generated force approached 4 N after 30 s, which is sufficient to move the piston since it is higher than the static friction force, as discussed above. Hence, it is confirmed that the amount of reagent was sufficient to create the necessary thrust to deliver ink into tissue.

### 3.3. Triggering Mechanism Validation

**Figure 7:**
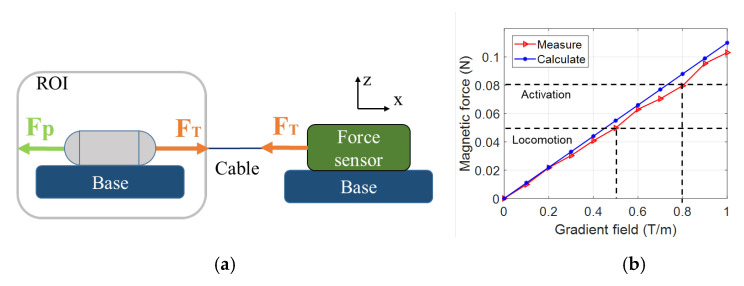
(**a**) Configuration setup to measure the magnetic force produced by the EMA system. (**b**) The results of the magnetic force and defined values for working modes of TCE.

The external electromagnetic field is crucial in the proposed TCE system to produce the driving force for capsule locomotion, the force for extruding and withdrawing the injection needle, and the activation force for the chemical reaction. As shown in Figure 7a, we carried out an experiment to investigate the magnetic force created by the EMA system. The TCE, which was positioned inside the ROI of the EMA system, was linked to a force sensor (Advanced Digital Force Gauges Series 5, Mark-10, Copiague, NY, USA) via a cable. To avoid the effects of the generated magnetic field, the sensor was installed outside the control system. We aligned the TCE along the *z*-axis at 10 mT magnetic field, and then the gradient magnetic field was used to pull the capsule along the *x*-axis at different levels, from 0 T/m to 1.2 T/m. Since the pulling force and string tension were equivalent, we could obtain the resultant magnetic force by acquiring the tension data. The analog signal at the sensor was collected at a sampling rate of 1 kHz. Figure 7b shows the magnetic force from measurements and theoretical calculations. We can see that the measurement values are close to the theoretical data, and the maximum magnetic force is approximately 0.1 N. Different levels of gradient field were used to perform different tasks.

**Figure 8:**
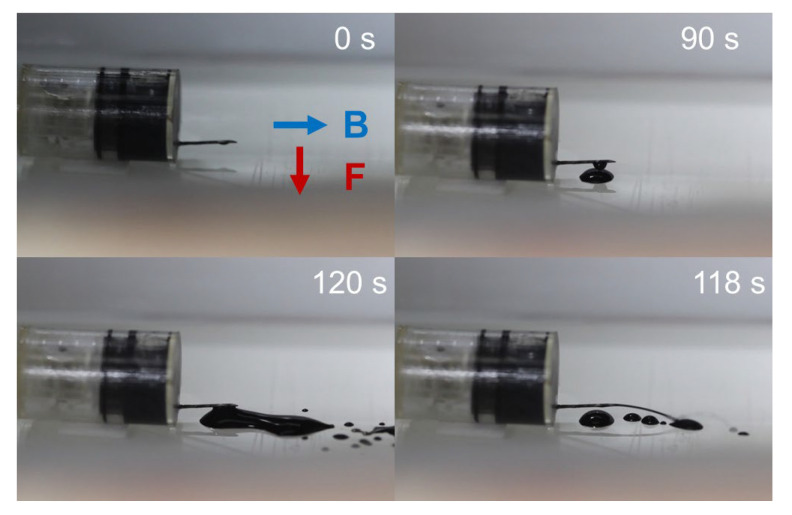
Validation of the triggering mechanism to release ink.

Next, we assembled the tattooing module with the following steps: (1) loading ink, (2) installing the piston, (3) loading chemical powder into the cavity of the piston, (4) adding the water box, (5) installing the magnet mechanism, and (6) sealing the open end. It is crucial to ensure that the surface of the propellant room is dry before loading the chemical powder. Then, we placed the module to the ROI of the EMA system to test the triggering function. A vertical direction magnetic field of 10 mT was used to align the magnet, and a vertically downward direction gradient field 0.8 T/m was generated to break the water reservoir and activate the mechanism. As shown in Figure 8, the ink started releasing after 90 s. Due to the ongoing creation of gas, the pressure inside the propellant room began to rise. When the force on the piston was higher than the static friction force between the piston and body, it was pushed at a significant force, creating an ink stream at the needle. It took roughly 120 s for the ink to be ejected. It was confirmed that the triggering mechanism can be remotely controlled by the external magnetic field.

### 3.4. The 5 DOF Locomotion

**Figure 9:**
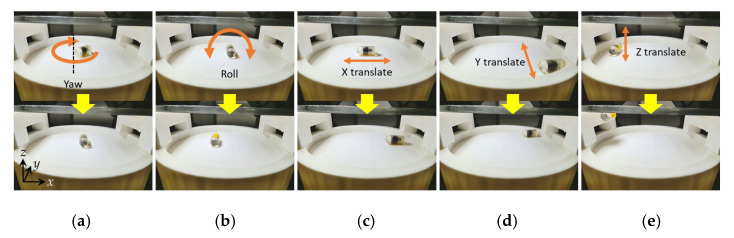
The 5 DOF locomotion of the TCE robot: (**a**) yaw motion; (**b**) roll motion (a yellow marker on capsule was used to recognize the roll motion); (**c**) *x*-axis translation; (**d**) *y*-axis translation; (**e**) *z*-axis translation.

To validate the controllability of the proposed TCE robot in 5 DOF, we moved the robot to an open-space plastic base. The capsule was loaded with ink and chemical reagents to evaluate the motions under friction and gravity force at a full load. Figure 9a,b show the 2 DOF rotation motion, including yaw and roll motion, which were used to scan or position the injection needle. These motions can be realized using a magnetic field with a magnitude of 10 mT. A low-value magnetic field can cause vibrations in the capsule’s pose due to the imbalance between the gravity force and magnetic torque. Figure 9c–e show the 3 DOF translation motions along the *x*-, *y*-, and *z*-axis. The translation motions were executed by creating a gradient field to pull the robot toward the high-density magnetic field region after it was aligned along the desired direction. A ratio of 10 between the gradient field and magnetic field was used as a control law. This ratio guarantees uniformity in the magnetic field and, thus, results in an accurate motion along the planned path. A 100 mT/m gradient field was used to move the TCE robot in the *xy* plane, and a higher value, 300 mT/m, was required to move the robot along the *z*-axis due to the gravity force. In this test, we demonstrated fundamental motions in 5 DOF of the developed TCE robot for the locomotion’s functionality. These motions can be combined to perform complex movements. In addition, we validated that the locomotion was completed at a lower gradient field level than that required for the activation function, and the ink injection was not activated during locomotion.

### 3.5. Ex Vivo Experiment in a Pig Small Intestine

**Figure 10:**
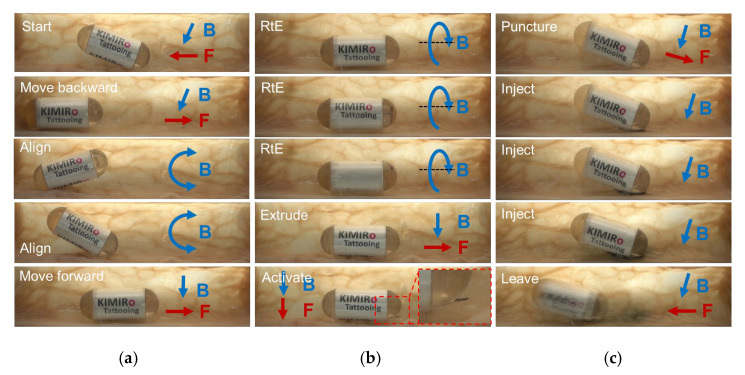
Experiment result of the proposed tattooing capsule endoscope in a segment of small intestine: (**a**) capsule locomotion; (**b**) needle extrusion; (**c**) tattooing and needle retraction. RtE means Rotate to Extrude. Blue and red arrows depict the magnetic field and force direction, respectively.

Finally, we performed a complete experiment to validate the locomotion and tattooing function of the proposed TCE. Figure 10 shows the results of the ex vivo experiment conducted in a pig’s small intestine segment. The small intestine was purchased from a local market, gently washed with water, and then fixed onto a tube that was cut in half for visualization. The first test involved basic movements for visual investigation. After the robot was aligned, the magnetic field gradient of 0.3 T/m was produced to move it. Figure 10a shows how a capsule could be maneuvered and transported within the gut without opening the injection needle. The tattooing function would not be activated during locomotion, since the gradient field required for the triggering mechanism is much higher than the locomotion gradient field (higher than 0.8 T/m, as discussed in Section 3.3). It is worth noting that the gradient field direction in locomotion is parallel to the axial axis of the capsule robot. The resultant force tends to push the magnet forward, so the likelihood of triggering the chemical reaction is small. The injection needle was then opened at the target by applying a rotating magnetic field about the capsule axial axis in a clockwise direction, followed by a magnetic force generating a forward capsule direction to extrude the needle, as shown in Figure 10b. The location of the capsule was steadily maintained during the needle extrusion process. Then, the chemical reaction was activated. This is needed to compress the helical spring 1.2 mm to allow for the microneedle to puncture the water reservoir. At this status, the reaction force of the compressed spring is approximately 0.06 N. A magnetic field gradient of 0.8 T/m was created in a downward direction, which can generate about 0.08 N, and the permanent magnet was pushed downward to break the water reservoir and squeeze the water. Ink injection is the next step, as seen in Figure 10c. Since the chemical reaction needs time to produce gas, we could utilize this time to insert the injection needle into the tissue. When the needle was inserted, the capsule had a 30° aligned incline. Due the tubular shape of the small intestine, this alignment motion could be performed by combining the yawing and rolling motions. A magnetic force was provided to penetrate the tissue using a high gradient field. After approximately 90–120 s, once the piston’s thrust exceeded 4 N, the ink was injected into the tissue. After that, the TCE was controlled to continue traveling and moving out.

## 4. Conclusions

In this study, we proposed a novel functional capsule endoscope that can actively travel in the digestive tract for visual diagnosis and can perform tattooing at the destination. The overall design of the system, consisting of the tattooing capsule endoscope and the EMA system, was discussed. We presented the design of the capsule robot using the tattooing mechanism. A prototype robot was fabricated with dimensions of 12.5 mm in diameter and 34.6 mm in length. The multifunctionality of the developed capsule robot was validated through locomotion and tattooing tests, which were both controlled by the EMA system. As a result, the proposed tattooing capsule was successfully implemented using the ex vivo experiment; thus, it is worth noting that the proposed capsule uses a battery-free activation mechanism to execute the medical function.

The proposed design has several limitations. First, the size of the capsule is still large compared to commercial WCEs due to the fabrication method. The capsule dimension could be reduced using advanced fabrication technologies. Second, the insertion of the injection needle into the tissue is challenging since there is no feedback information regarding the angle between the TCE and the organ’s surface. In this work, we assumed that the environment is known, with the surface parallel to the xy plane, so the insertion angle is the alignment of the TCE. For in vivo applications, this information has to be investigated. Third, the proposed mechanism cannot perform multiple tattooing since once the chemical reaction is activated, it cannot be reused. Therefore, future work will include developing a tracking system to detect the capsule’s position and orientation. The tracking data can be combined with the camera’s visual information to estimate the surface’s slope using the computer vision technique. In this way, the operator can easily and accurately insert the needle into the tissue. A multiple tattooing mechanism will also be considered.

## Figures and Tables

**Table 1:** Specifications of the EMA system.

Coil	Mx 1	Mx 2	RC 1, RC 2	HH
Type	Maxwell	Maxwell	Rectangular	Helmholtz
Radius (mm)	195	100	n/a	195
Width × Length (mm)	n/a	n/a	156 × 337	n/a
Distance (mm)	337.75	173.2	200	195
Coil turns	1426	660	600	710

**Figure A1:**
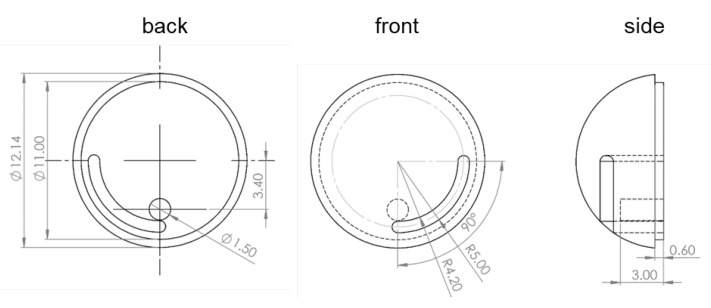
Drawing of the head dome.

## Data Availability

Not applicable.
